# GPCRsignal: webserver for analysis of the interface between G-protein–coupled receptors and their effector proteins by dynamics and mutations

**DOI:** 10.1093/nar/gkab434

**Published:** 2021-06-01

**Authors:** Przemysław Miszta, Paweł Pasznik, Szymon Niewieczerzał, Jakub Jakowiecki, Sławomir Filipek

**Affiliations:** Faculty of Chemistry, Biological and Chemical Research Centre, University of Warsaw, 02-093 Warsaw, Poland; Faculty of Chemistry, Biological and Chemical Research Centre, University of Warsaw, 02-093 Warsaw, Poland; Faculty of Chemistry, Biological and Chemical Research Centre, University of Warsaw, 02-093 Warsaw, Poland; Faculty of Chemistry, Biological and Chemical Research Centre, University of Warsaw, 02-093 Warsaw, Poland; Faculty of Chemistry, Biological and Chemical Research Centre, University of Warsaw, 02-093 Warsaw, Poland

## Abstract

GPCRsignal (https://gpcrsignal.biomodellab.eu/) is a webserver devoted to signaling complexes of G-protein–coupled receptors (GPCRs). The recent improvement in cryo-electron microscopy resulted in the determination of a large number of high-resolution structures of GPCRs bound to their effector proteins: G proteins or arrestins. Analyzing the interfaces between receptor and an effector protein is of high importance since a selection of proper G protein or specific conformation of arrestin leads to changes of signaling that can significantly affect action of drugs. GPCRsignal provides a possibility of running molecular dynamics simulations of all currently available GPCR-effector protein complexes for curated structures: wild-type, with crystal/cryo-EM mutations, or with mutations introduced by the user. The simulations are performed in an implicit water-membrane environment, so they are rather fast. User can run several simulations to obtain statistically valid results. The simulations can be analyzed separately using dynamic FlarePlots for particular types of interactions. One can also compare groups of simulations in Interaction frequency analysis as HeatMaps and also in interaction frequency difference analysis as sticks, linking the interacting residues, of different color and size proportional to differences in contact frequencies.

## Graphical Abstract

**Graphical Abstract:**
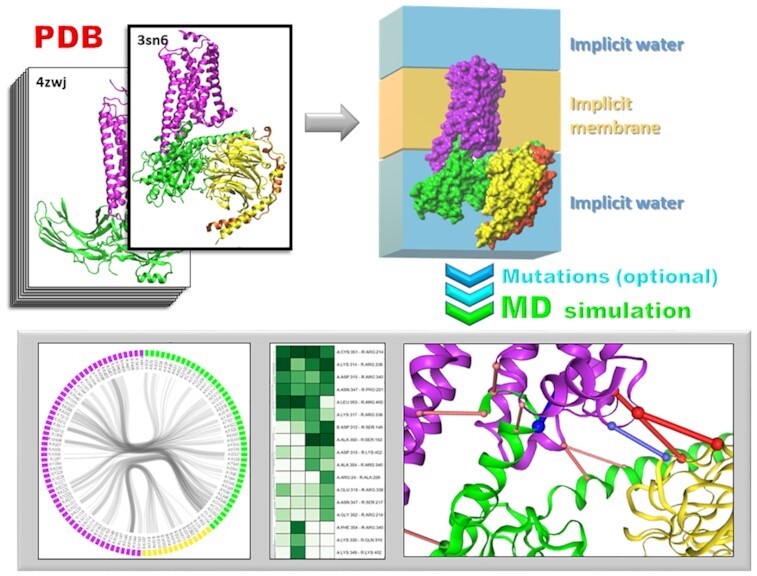
The main features of GPCRsignal.

## INTRODUCTION

G-protein–coupled receptors (GPCRs) constitute the largest family of cell membrane receptors. It comprises of >800 members which mediate majority of physiological functions in human signaling systems such as the nervous, muscular, skeletal, endocrine and digestive systems among others (1,2). Since GPCRs are the essential cell membrane signaling molecules and malfunctions of GPCRs cause severe diseases, they represent the most important drug targets—it is estimated that one-third of all currently used drugs are directed toward GPCRs (3). Structurally, the cores of all GPCRs are very similar: extracellular N-terminus, a bundle of seven transmembrane α-helices (TMs) with extracellular and intracellular loops and intracellular C-terminus. There are several classes of GPCRs differing mostly in size and function of extramembrane domains. According to the GRAFS classification system (4), the human GPCRs are divided into five families: the rhodopsin-like (class A), secretin (class B1), adhesion (class B2), glutamate (class C) and the frizzled/taste 2 family (class F); however, the newest data (5) suggest that there are more families (sometimes with only one representative receptor) and that frizzled and taste 2 form separate families. Most receptors (about 700) belong to class A that has a main (orthosteric) ligand-binding pocket between the transmembrane helices.

GPCRs were long thought as being localized only in the plasma membrane and allowing transmitting of the external signals into the cell interior. It is now recognized that GPCRs signal also at various intracellular locations, and the mechanisms and (patho)physiological relevance of such signaling modes are being actively investigated. Activation of GPCRs is accompanied by the outward movement of the intracellular part of transmembrane helix TM6 and also a smaller movement of helix TM5, which creates a cavity on the cytoplasmic side of the TM domain to bind G protein. Because of large excess of GPCR types over G proteins, it has been thought that every GPCR couples to a specific G protein subfamily; however, there is an experimental evidence that GPCRs can activate G proteins from multiple families. After phosphorylation of the C-terminus of GPCRs by G-protein–coupled receptor kinases (GRKs) arrestin can bind and block G protein coupling, but at the same time it participates in the second route of signaling (6,7). A tendency of GPCRs to work through different signaling modes is largely based on the structural plasticity of the receptors themselves and also in complexes with effector proteins (8). Most ligands of GPCRs can activate both routes of signaling (unbiased ligands), but some ligands preferentially activate only a subset of the signaling pathways (biased ligands) (9,10). That is why they have a great potential to become next-generation GPCR drugs with less side effects due to preferable activation of desired signaling pathways (11). Biased and unbiased signaling are extremely important in pharmacology, and the structures of GPCRs with G proteins and arrestins can help elucidate details of such signaling (12).

GPCRs can sense a large number of extracellular stimuli such as neurotransmitters, nucleotides, peptides, small proteins, lipids, as well as photons and ions, and translate that external signal into cellular responses by activating intracellular effector proteins: heterotrimeric G proteins and arrestins. G proteins are organized in four different families (G_s_, G_i/o_, G_q/11_ and G_12/13_) and comprise a total of 16 distinct subfamilies based on their Gα subunits (13). After activation, GPCRs are desensitized through phosphorylation and subsequent binding of β-arrestin, which can lead to receptor internalization. β-Arrestins can act as scaffolds for other proteins such as kinases and phosphodiesterases among others (10,14). Moreover, in recent years it has been found that β-arrestin can bind to GPCRs in at least two distinct conformations and the distinct GPCR–arrestin complexes can lead to distinct cellular functions (15–18). The arrestin family comprises of visual arrestins, β-arrestins and α-arrestins. β-Arrestins belong to a family of multifunctional scaffolding proteins. Functions of α-arrestins are less known than other arrestins, but they can serve as versatile adaptors that link GPCRs or the Notch receptor to E3 ubiquitin ligases and endocytic factors. The α- and β-arrestins could also form transient heterodimers that form a bridge between cargo and E3 ubiquitin ligases for subsequent trafficking (19).

Currently, there are about 100 structures of GPCRs determined in a complex with an arrestin or a G protein. The preferable method for structure determination of such complexes is cryo-EM since these complexes can be very large including stabilizing antibodies/nanobodies and lipids surrounding GPCR in a micelle or nanodisc. However, the first structure of GPCR with effector protein was determined by X-ray diffraction of a crystal—it was the β_2_-adrenergic receptor bound to G_s_ (20). In recent years (2019–2020), there has been an increase with over 200% in the number of structures of GPCR complexes with effector proteins and a similar pace is expected. Some unusual structures have also been determined, like the fungal class D receptor dimer coupled to two G proteins (21). Another example is the structure of the adrenomedullin receptor complex with G protein and with RAMP (receptor activity-modifying protein) (22). Because of those and other structures, a new service combing the functionality of a curated database and a web server would be highly beneficial to analyze structures of the interfaces of GPCR-effector protein complexes. It is needed to elucidate effects of particular residues on the effector protein selectivity and biased signaling. This can be done by mutation of these residues and analysis of the changes in the interfaces. One can also make simulations of wild-type structures as well as with engineered mutations introduced for structure stabilization.

There are two web servers offering MD simulations for GPCRs: GPCR-ModSim (http://gpcr-modsim.org/) and Hybrid MM/CG (https://mmcg.grs.kfa-juelich.de/). The GPCR-ModSim (23) was created for homology modeling of GPCRs, and the constructed model is equilibrated for 5 ns in explicit all-atom water-membrane environment using a hexagonal periodic box. The POPC (palmitoyl-2-oleoyl-sn-glycero-3-phosphocholine) membrane and the SPC water model are used. The OPLS force field is employed for the protein atoms, while the Berger parameters are used for the membrane. In the Hybrid MM/CG webserver (24), there is a possibility to run a combined all-atom and coarse-grained MD simulation, up to 10 ns, of GPCR-small ligand complex. The part of the protein with the ligand bound is simulated in all-atom representation in water hemisphere (MM) while the rest of protein in coarse-grained representation (CG). For MM part, the Amber14SB force field is used for protein atoms, TIP3P model for water and GAFF force field for small molecule ligands. The CG region is described by Gö-like potential. In both servers, there is a possibility to introduce mutations by submitting the manually modified GPCR sequence (in Hybrid MM/CG via GOMoDo web server (25)); however, none of them is dealing with complexes of GPCRs with effector proteins. None of them is also analyzing MD simulations for particular residue–residue interactions (via FlarePlots or HeatMaps). There is also no possibility to compare series of simulations.

The GPCRsignal allows to run only the curated complexes of GPCRs with effector proteins, so it is also related to databases on GPCRs: GPCRdb (https://gpcrdb.org/) and GPCRmd (https://submission.gpcrmd.org/home/). The GPCRdb database (26) is based on the static structures of GPCRs and their complexes with no ability to introduce mutations to the structure and analyzing their dynamic effects. However, GPCRdb has many tools for analysis of mutations and to design mutations for the ligand-binding site (ligand-site mutation design tool), thermostabilizing mutations (construct design tool) and G-protein–coupling altering mutations. The mutation design tools has been updated with the new mutagenesis data and receptor–ligand interactions from structures. The other related great web resource is GPCRmd (27). That service is a database for analyzing deposited trajectories of all-atom MD simulations performed in explicit membrane and water of curated GPCR–ligand complexes. However, there is no possibility to introduce mutations and running simulations online. The GPCRmd is focused on receptors, so it does not contain complexes of GPCRs with effector proteins.

The GPCRsignal web server has many features which can be used by a broad range of researchers and students, molecular modelers as well as experimentalists. The service is friendly to be used by unexperienced people and the extensive tutorial explains the procedures and analyses in detail and also provides a graphical introduction to the signaling processes of GPCRs.

## MATERIALS AND METHODS

### Web interface

The web interface was created in Bootstrap/jQuery/HTML5 with protein visualization Javascript libraries: Mol* (https://github.com/molstar/molstar) (28), MolArt (29), NGL Viewer (30,31) and some python visualization tools: Flareplot (time-flare version) (https://gpcrviz.github.io/flareplot/), get_contacts (https://getcontacts.github.io/) for the Interaction frequency analysis. We used AJAX loading for bigger and on-the-fly computed elements and AJAX updated status of progress bar while computing main job.

### Backend of the server

The server is developed for running one machine with Django framework hosted web interface, maintaining jobs and serve database of complexes and multiple GPGPU servers for running user jobs. Our infrastructure is built on nodes containing two GPUs each, so we decided to run two user jobs per node and share our resources depending on final server usage to minimize waiting time. The server will stay free for all users. The calculations are relatively fast, so one can perform series of MD simulations. Longer MD simulations are also possible but off the web server on the individual basis since the server and underlying methodology are in constant development. Molecular dynamics process is performed in implicit water and membrane environment using IMM1 (32) method implemented in NAMD v.2.13 (33) running on CUDA-enabled GPUs server.

### Implicit environments calculation

**Figure 1.**
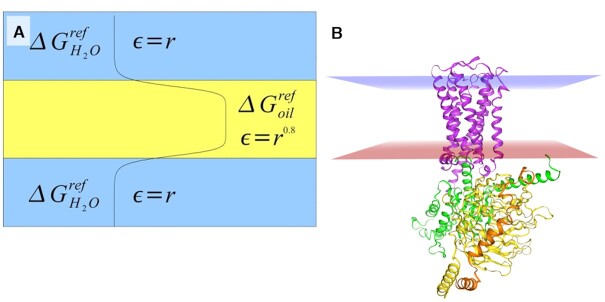
An implicit solvent method IMM1. (**A**) A continuous change of solvation potential in a water-membrane system. (**B**) A GPCR in the implicit membrane environment.

Molecular dynamics simulations of GPCRs in complexes with effector proteins and peptide ligands are executed in the service based on the all-atom representation of protein structures embedded in the implicit heterogeneous environment representing both solvent and membrane media. The approach is based on the implicit membrane methodology IMM1 (32) being the extension of EEF1 method (34). The method is defined only on CHARMM19 polar force field combined with a Gaussian model for the solvation free energy. The implicit solvation term has a form:

$$\begin{equation*}\Delta {G^{solv}} = \mathop \sum \limits_i \Delta G_i^{solv} = \mathop \sum \limits_i \Delta G_i^{ref} - \mathop \sum \limits_i \mathop \sum \limits_{j \ne i} f_i^{free}\left( {{r_{ij}}} \right){V_j}\end{equation*}$$

where $\Delta G_i^{solv}\;$is the solvation free energy of atom *i*, and ${r_{ij}}$ is a distance between atoms *i* and *j*, $\Delta G_i^{ref}$ is free energy of atom *i* in a small group exposed to solvent, $\;{V_j}$ is a volume occupied by atom *j*, and $f_i^{free}$ is the free energy density of atom *i*. The solvation model takes into account how neighboring atoms affect the solvation energy of a given atom ($\Delta G_i^{ref}$) by excluding solvent from the surrounding space. Free energy density has a form of Gaussian function. The membrane is introduced as a slab parallel to *xy* plane centered at *z* = 0. The solvation parameters for each atom are defined for both environments (solvent and the membrane), and they smoothly transit at the interface using the switching function. Figure 1 shows a scheme of a continuous change of solvation potential in a water-membrane system and also a GPCR simulated in implicit membrane environment.

The transition region between the hydrophobic core and the solvent environment is about 6 Å wide, which is in agreement with experimental data of lipid bilayers. The electrostatic interactions are modeled by distant dependent dielectric constant. For details of the implicit solvent method, please see the original papers (32,34). For simulations, we use Langevin dynamics with damping constant of 40 ps^–1^ and with 2 fs time step whereas all bond lengths are constrained using SHAKE (35) algorithm. The simulations run at temperature 298 K. Originally, the IMM1 method was implemented in CHARMM program (36); however, herein our implementation of the method in NAMD (33) is used to facilitate from GPGPU and parallelization. CHARMM19 force field is used since the IMM1 method was parametrized only for this force field. CHARMM19 is a united-atom force field without aliphatic hydrogens, which additionally diminishes number of atoms in the system. CHARMM19 was parametrized only for standard amino acids so to study interactions of arrestins with phosphorylated residues on C-terminus of GPCRs we made parametrization of phosphorylated serine (SEP, ‘s’ in one-letter code for flareplots and mutations) and threonine (TPO, ‘t’ in one-letter code for flareplots and mutations). Because of lack of parametrization of small molecules in CHARMM19, all ligands except the peptide ones had to be removed from calculations (such information has been placed in MD&mut page close to ‘Start MD’ button); however, because of short length of simulations such influence is negligible. We plan to upgrade to all-atom CHARMM36 force field where there are parameters for small ligands to use in explicit environments. New parameters, specific for implicit environments, for small ligands and also for amino acids, will be developed and validated.

### Curation process of structures

**Figure 2.**
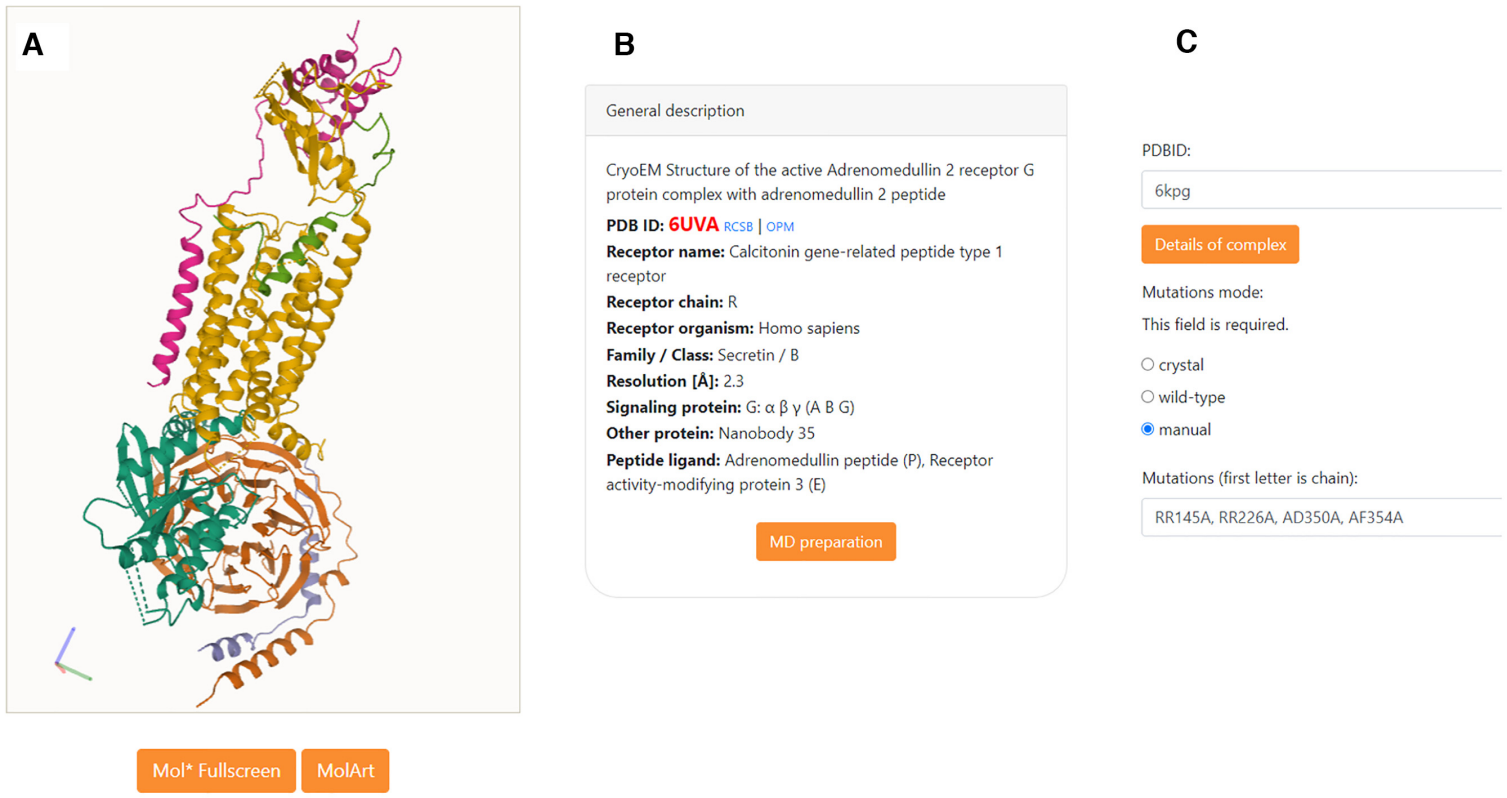
(**A**) Structure of selected PDB ID: 6UVA complex in Mol* viewer. One can also see it in MolArt viewer. (**B**) One of tiles describing details of the complex. (**C**) MD&mutation preparation page filled with data from example input (CB_1_ cannabinoid receptor PDB ID: 6KPG).

During curation process all antibodies/nanobodies and fusion proteins were removed. Nonpeptide ligands were also removed due to usage of CHARMM19 force field. The peptide ligands and also some additional proteins, like the receptor activity-modifying protein (Figure 2A,B), are kept and included in simulations. In case of dimeric structures (currently, there is one such structure PDB ID:7AD3), all dimers are kept. Since one of G proteins is not visible in that experimental structure we made a model of a full dimeric complex which is available with a new accession name, 7AD3m. This dimeric complex is also available for mutations and simulations.

Single residues lacking a certain number of atoms were rebuilt; however, the lacking ranges of residues in loops and also in N- and C-termini were not restored. The ends of lacking fragments were left uncharged during preparation of input structures for simulations. The missing fragments in experimental structures will be reconstructed in the next version of the web server. The smaller fragments will be wholly rebuilt while the larger fragments will be substituted with smaller ones. However, this should be done with caution. Two lists of complexes will be available: without modifications and with missing fragments restored. Reconstruction of part of receptor’s C-terminus is also regarded as it was found important for G protein binding and especially for arrestin binding (in this case with phosphorylated residues). For each complex in our service two structures were prepared: the structure with engineered mutations and wild-type (WT) structure. The user defined mutations, also to and from phosphorylated serine ‘s’ and threonine ‘t’, are introduced to WT structure and the simulation is started.

### Contact network analysis

GetContacts software is used for analysis of the interface interactions between the receptor and the effector protein. Flareplot (https://gpcrviz.github.io/flareplot/) is used to visualize the interface interactions occurring in a user-selected timeframe of the trajectory. In our web server, using get_dynamic_contacts.py tool, we create a list of all residue–residue contacts between the receptor and the effector protein occurring in each frame of the MD trajectory. Next, using get_contact_frequencies.py module, we calculate the frequency of each interaction found in the contact list file. The interaction frequency (Freq) ranges between 0 and 1: Freq = 1.0 means that a particular interaction is present in every single trajectory frame, Freq = 0.5 means the interaction is found only in 50% of the frames, while Freq = 0 means the interaction does not occur in the analyzed trajectory.

In order to identify those contacts for which the frequency differs significantly between the two selected groups of simulations, we calculate the difference between average frequencies of a particular contact (Δ*_freq_*, described by equation below):

$$\begin{equation*}{\Delta _{{\rm freq}}} = \overline {Fre{q_{{\rm group}\;1}}} {\rm{\;}} - {\rm{\;}}\overline {Fre{q_{{\rm group}\;2}}} \end{equation*}$$

If the absolute value of the difference between the average frequencies of a particular contact calculated for the two sets of trajectories is higher than a specific cut-off value, then the contact is kept as a potentially meaningful (e.g. if cutoff is set to 0.4, then only the contacts with |Δ*_freq_*| > 0.4 are kept). We visualize the average frequency differences for these meaningful interactions as red and blue sticks linking C_α_ atoms of the interacting residues.

### Workflow

We have created user-job pipeline with four steps and additional calculations on-the-fly while loading page or selecting elements by user. On-the-fly mode applies to initial loading of Interaction frequency analysis (HeatMaps) and to the Interaction frequency difference analysis (Sticks). By changing the contact type (hydrogen bonds, salt bridges and aromatic interactions) and/or cutoffs for HeatMaps and Sticks they are recalculated and user can check status messages (computing/done/error). Server is importing data from internal GPCRsignal database, such as PDB and PSF files, membrane thickness, and chain identifiers for receptor and each effector protein.

The steps are:

1. Preparation phase—applying mutations to WT structure and preparing NAMD input files.
2. Molecular dynamics—running all-atom MD simulation in implicit environments.
3. Plotting—running get_contacts scripts (computing for displaying results and pre-computing as much as possible for on-the-fly computations).
4. Finishing – creating archives, copying working files to results directory, sending e-mail with job status.

## RESULTS

### Description of input

User can browse through a table of curated structures of GPCR-effector protein (G protein or arrestin) complexes. After selecting a structure, the user can go into details of the complex presenting structure and descriptions on several tiles (Figure 2A,B) or go to the MD&mutation preparation page (Figure 2C).

In the MD&mutation page the Mutations mode (Figure 2C) has to be selected: (i) with crystal/cryo-EM structure mutations, (ii) WT or (iii) WT with user defined mutations. There is no maximal number of user mutations. Then, parameters of simulations are to be specified or their default values used: (i) total length of a simulation: default = 10 ns, min = 5 ns, max = 25 ns; (ii) number of frames in a simulation: default = 100, min = 5, max = 200; and (iii) the membrane thickness: default value is taken from internal database (based on value for particular receptor in OPM database (37) ), min = 20 Å, max = 40 Å. All the above selections must be confirmed by the user after pushing ‘Start MD’ button. On the confirmation page, there is also a possibility to add a job description and e-mail for receiving notification of job completion.

### Description of output

**Figure 3.**
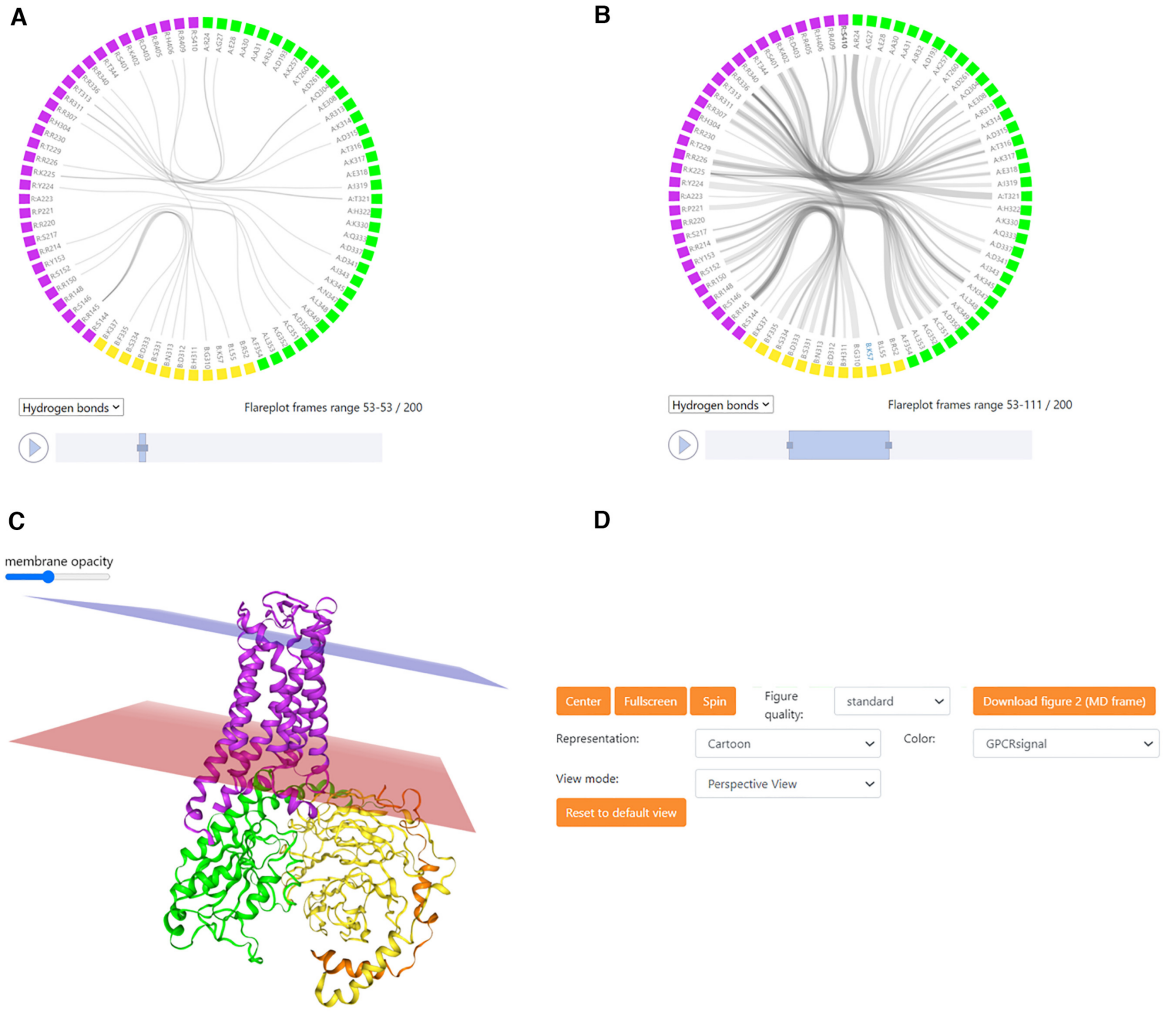
Analysis of a single trajectory. (**A**) FlarePlot showing contacts in the interface between receptor (purple color) and effector protein (G_α_—green, G_β_—yellow, G_γ_—orange) for the single frame. (**B**) The same for range of frames. (**C**) The structure of the complex with blue and red transparent squares visualizing the edges of the membrane. (**D**) The menu for displaying a complex structure. The color mode GPCRsignal is the same as shown in panel (C).

After completion of the MD simulation a new page starts with analysis of single trajectory. On that page one can select type of contact, e.g. hydrogen bonds, and the FlarePlot can visualize contacts in the interface between receptor and effector protein for the single frame (Figure 3A), range of frames (Figure 3B). The structure of the complex (Figure 3C) is changing as the simulation is playing and it refers to a particular frame or the first frame if a range of frames is selected. A variety of options for displaying a structure are available (Figure 3D).

**Figure 4.**
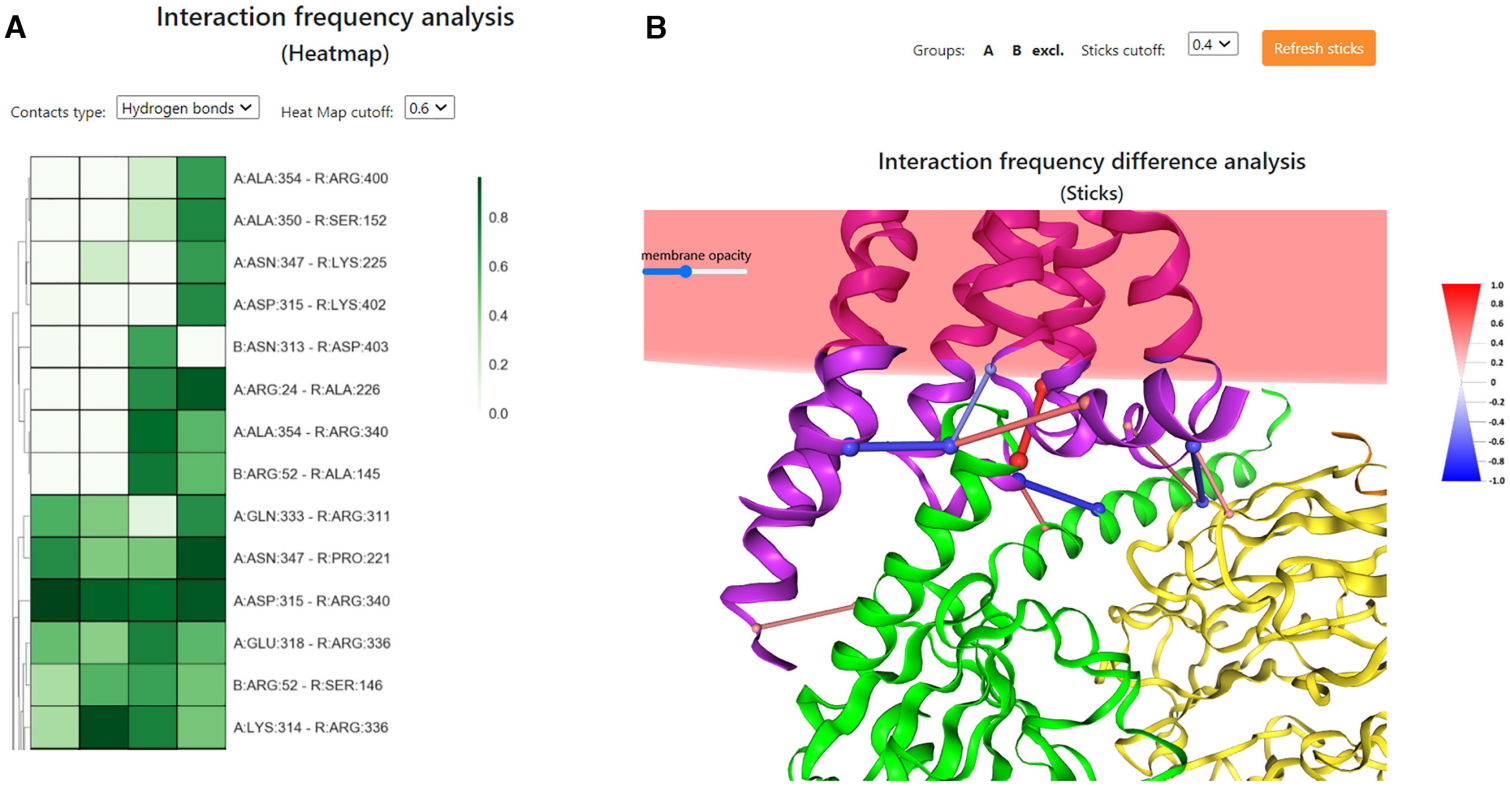
(**A**) Interaction frequency analysis on HeatMaps. A scale bar shows percentage of frequencies. A cutoff excludes the smallest contact frequencies. The first two simulations are for WT, while two others for mutated structure. (**B**) Interaction frequency difference analysis for selected two groups of simulations denoted A and B. Here, group A is for WT simulations and B for simulations with mutated structure. The sticks linking C_α_ atoms of interacting residues are colored in red and blue meaning positive or negative difference in contact frequencies, respectively. The size of sticks and color intensity are proportional to particular contact frequency difference and is visualized on a scale hourglass. As before, the cutoff excludes the smallest differences in contact frequencies.

Analysis of multiple MDs starts from selecting of completed simulations by copying their tokens into a table on ‘Comparison of multiple MDs’ page. The computed trajectories are stored on the server for two months. Comparison of trajectories can be done via Interaction frequency analysis on HeatMaps (Figure 4A) and via the Interaction frequency difference analysis (Figure 4B). In both cases, the specific cutoffs can be set up to exclude the smallest contact frequencies. For the latter analysis, the interaction frequency difference is visualized via sticks linking interacting residues (their C_α_ atoms) with blue and red color meaning positive or negative difference in contact frequencies. Since the simulations start each time with different set of atom velocities the final structures and resulting contacts can be different even for the same initial complex structure as this is a statistical process. Therefore, a series of shorter simulations with later averaging is preferred over one longer simulation.

One can download a package with results for each task. It is a ZIP file including basic files: protein structure (format PDB/PSF), result of molecular dynamics (format DCD), job information file (text file), contacts files (format: tab-separated values) and Flareplot files (format json). Protein files are ready to use with corresponding DCD file if user want to visualize simulation in external visualization software. Protein results files depend on selected wild-type or crystal/cryo-EM structure mode, and include user applied mutations if they were used. From the job information file, one can take the job parameters or take a link to results to visualize those results on GPCRsignal server and make analyses (if within 2 months).

For the contacts, we supply file describing all contacts between receptor and effector proteins in simulation, and type-separated contact frequencies files. Flareplots could be generated by uploading those json files on the page: https://gpcrviz.github.io/flareplot/?p=create. It is useful when one needs to display results after 2 months (when data will be removed from our server) or when user want to make any changes to circular plot.

There is a possibility to share the selected MD simulations with other users. This can be done with a button ‘Make this job available to public’ on the results page (accessible just after the simulation is completed or later with the simulation token). The shared simulations are visible via menu item ‘Shared jobs’. The shared data can be analyzed as single trajectories or groups of simulations via ‘Comparison’ page.

### Performance and comparison to all-atom explicit system

MD simulation of 25 ns for CB_1_ receptor with trimeric G protein (PDB ID: 6KPG), with number of atoms and united atoms in CHARMM19 force field about 8700, takes about 6 h. For comparison, 25 ns MD simulation of the same system in full POPC membrane and water, containing about 120 000 atoms, takes about 120 h (5 days) on the same server.

**Figure 5.**
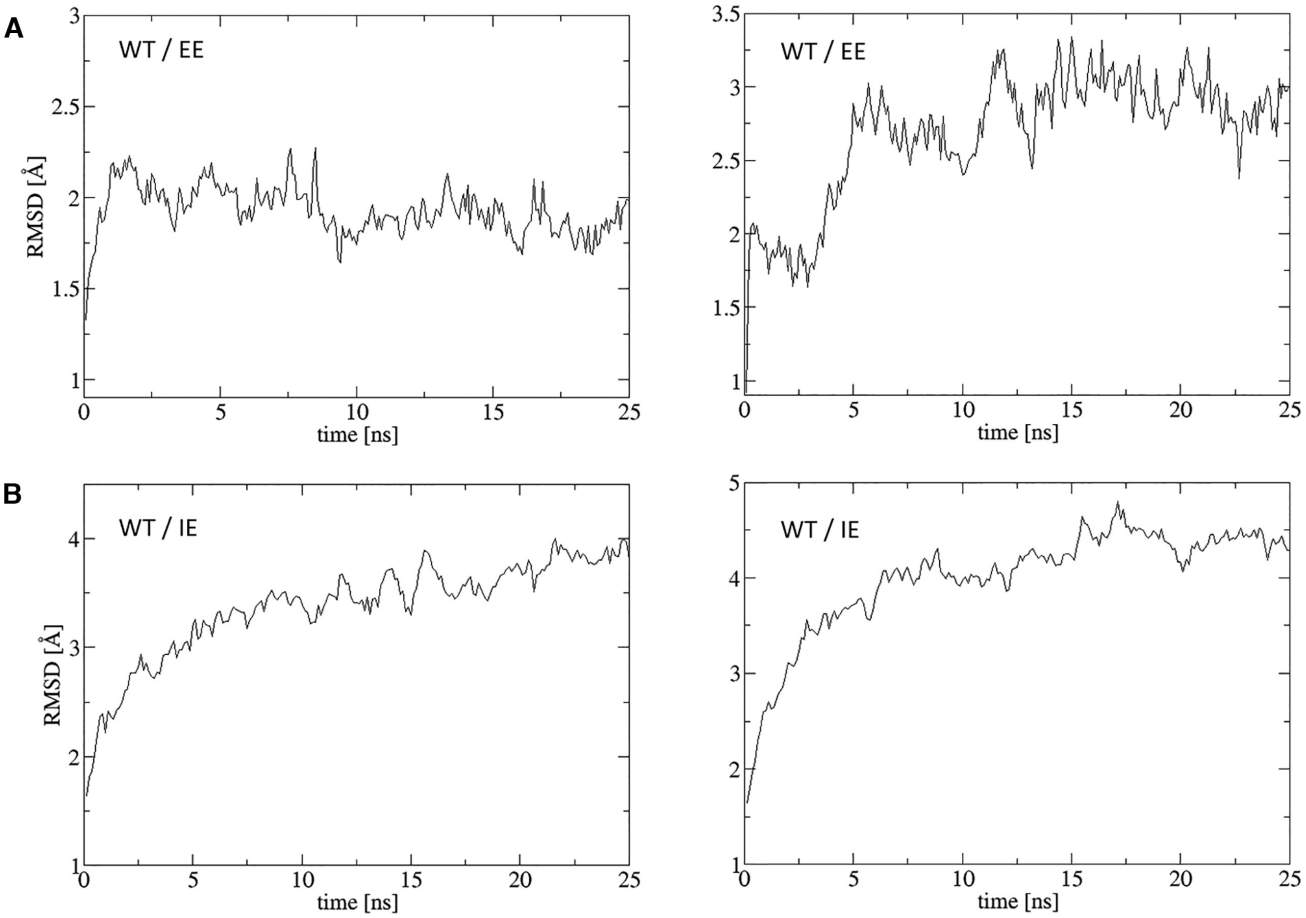
RMSD plots for the same exemplary wild-type (WT) CB_1_-G_i(αβγ)_ complex (PDB id:6KPG) simulated in explicit (**A**) and implicit (**B**) environments.

**Figure 6.**
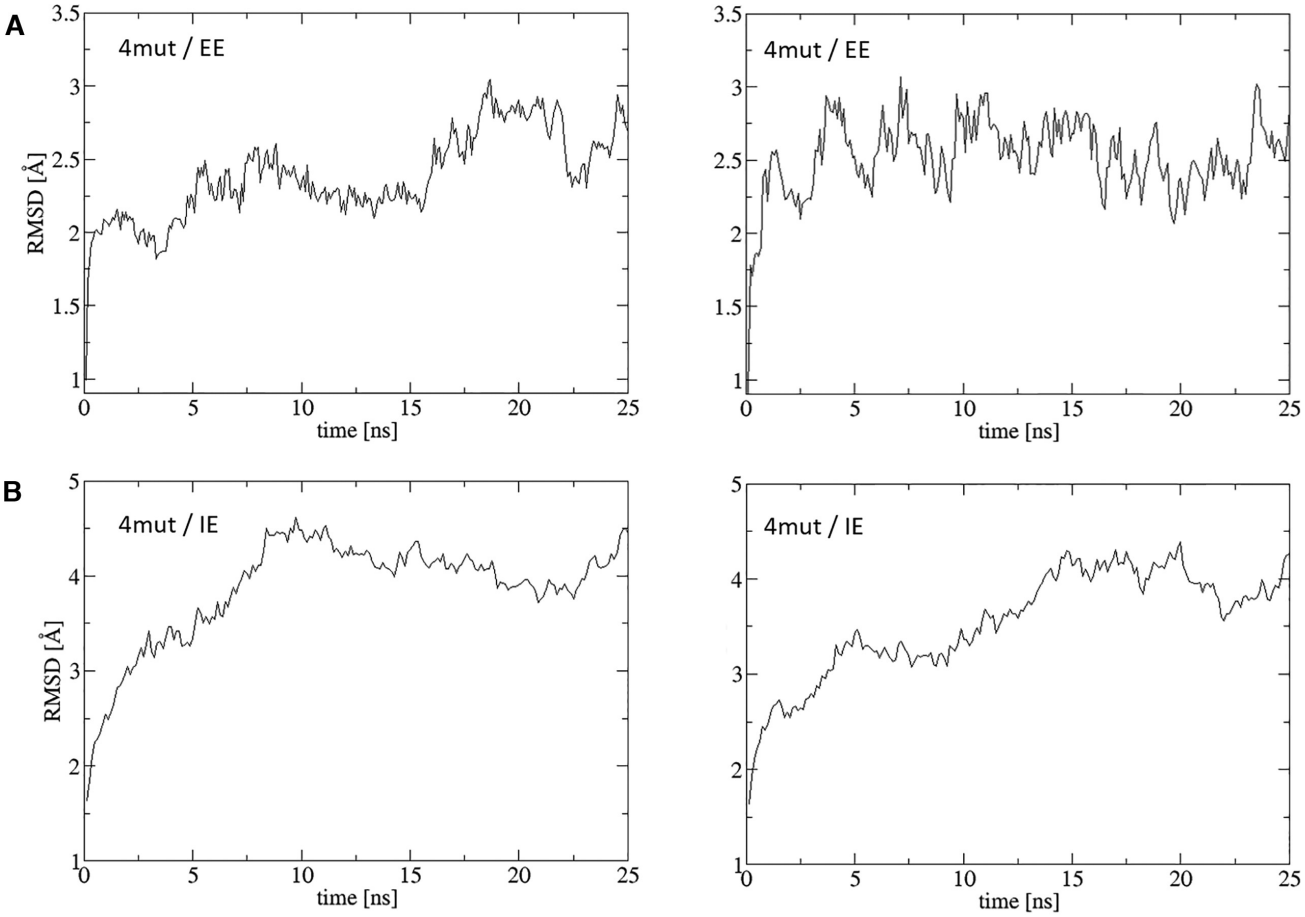
RMSD plots for the same exemplary CB_1_-G_i(αβγ)_ complex (PDB ID: 6KPG) with four user defined mutations (4 mut) simulated in explicit (**A**) and implicit (**B**) environments.

We compared RMSD (root-mean-square deviation) plots for exemplary complex PDB ID: 6KPG simulated in explicit (EE) and implicit (IE) environments (Figure 5). RMSD values were calculated for C_α_ atoms of the whole complex. For the WT complex simulated in EE the structure stabilized at 2 Å in one MD simulation but at 3 Å in the second simulation (Figure 5A) while it reached 4 and 4.5 Å in IE (Figure 5B). For the mutated complex simulated in EE, the structure stabilized at about 2.5 Å in both simulations (Figure 6A) and in IE at 4 Å (Figure 6B). For the mutated structure, we selected a set of disruptive mutations: R:R145A, R:R226A, A:D350A and A:F354A, where the first letter denotes a chain: R for receptor and A for G_α_ subunit. Such mutations disrupted salt bridges and stacking interactions but MD simulations conducted both in EE and IE show that the overall structure of the complex is still very stable. Local changes can be visualized using FlarePlots and HeatMaps in our server. Higher values of RMSD plots obtained in IE are a consequence of higher flexibility of the complex in implicit environments. Length of 25 ns of MD simulation seems to be adequate to obtain stability of complex structure, however, to compare the immediate effects of mutations much smaller MD simulations can be applied.

### Tutorial

The extensive tutorial starts from a slide show showing the GPCR signaling cycle which could be useful for students and also researchers not working routinely with GPCRs. Then, each page of the web server is described with all available options. The example output is also shown for analysis of single MD simulation (menu: Example 1) and for analysis of multiple MD simulations (menu: Example 2). All types of performed analyses are described and especially those for groups of simulations: the interaction frequency analysis (HeatMaps) and the interaction frequency difference analysis (Sticks).

## CONCLUSION

The GPCRsignal web server is intended to be used for analyses of the interface between GPCRs and their effector proteins: G proteins and arrestins. The analyses are performed in a dynamic way by MD simulations and mutations and they can be useful for researchers and students interested in GPCR signaling processes. Currently, there are about 100 structures of GPCR-effector protein complexes in the Protein Data Bank but due to enormous progress in experimental determination of such complexes we can expect a large number of such structures which will greatly facilitate research on biased signaling.

## DATA AVAILABILITY

GPCRsignal is free to all users without a login requirement. The e-mail is optional (for receiving notification of job completion) and is not stored on the server.

## References

[1] Rockman H.A. , Koch W.J., Lefkowitz R.J. Seven-transmembrane-spanning receptors and heart function. Nature. 2002; 415:206–212. 11805844 10.1038/415206a

[2] Hilger D. , Masureel M., Kobilka B.K. Structure and dynamics of GPCR signaling complexes. Nat. Struct. Mol. Biol. 2018; 25:4–12. 29323277 10.1038/s41594-017-0011-7 PMC6535338

[3] Hauser A.S. , Attwood M.M., Rask-Andersen M., Schioth H.B., Gloriam D.E. Trends in GPCR drug discovery: new agents, targets and indications. Nat. Rev. Drug Discov. 2017; 16:829–842. 29075003 10.1038/nrd.2017.178 PMC6882681

[4] Fredriksson R. , Lagerstrom M.C., Lundin L.G., Schioth H.B. The G-protein-coupled receptors in the human genome form five main families. Phylogenetic analysis, paralogon groups, and fingerprints. Mol. Pharmacol. 2003; 63:1256–1272. 12761335 10.1124/mol.63.6.1256

[5] Nordstrom K.J. , Sallman Almen M., Edstam M.M., Fredriksson R., Schioth H.B. Independent HHsearch, Needleman–Wunsch-based, and motif analyses reveal the overall hierarchy for most of the G protein-coupled receptor families. Mol. Biol. Evol. 2011; 28:2471–2480. 21402729 10.1093/molbev/msr061

[6] Gurevich V.V. , Gurevich E.V. GPCR signaling regulation: the role of GRKs and arrestins. Front. Pharmacol. 2019; 10:125. 30837883 10.3389/fphar.2019.00125 PMC6389790

[7] Seyedabadi M. , Gharghabi M., Gurevich E.V., Gurevich V.V. Receptor-arrestin interactions: the GPCR perspective. Biomolecules. 2021; 11:218. 33557162 10.3390/biom11020218 PMC7913897

[8] Sutkeviciute I. , Vilardaga J.P. Structural insights into emergent signaling modes of G protein-coupled receptors. J. Biol. Chem. 2020; 295:11626–11642. 32571882 10.1074/jbc.REV120.009348 PMC7450137

[9] Kenakin T. Biased receptor signaling in drug discovery. Pharmacol. Rev. 2019; 71:267–315. 30914442 10.1124/pr.118.016790

[10] Smith J.S. , Lefkowitz R.J., Rajagopal S. Biased signalling: from simple switches to allosteric microprocessors. Nat. Rev. Drug Discov. 2018; 17:243–260. 29302067 10.1038/nrd.2017.229 PMC5936084

[11] Bock A. , Bermudez M. Allosteric coupling and biased agonism in G protein-coupled receptors. FEBS J. 2021; 288:2513–2528. 33621418 10.1111/febs.15783

[12] Erlandson S.C. , McMahon C., Kruse A.C. Structural basis for G protein-coupled receptor signaling. Annu. Rev. Biophys. 2018; 47:1–18. 29498889 10.1146/annurev-biophys-070317-032931

[13] Milligan G. , Kostenis E. Heterotrimeric G-proteins: a short history. Br. J. Pharmacol. 2006; 147(Suppl. 1):S46–S55. 16402120 10.1038/sj.bjp.0706405 PMC1760735

[14] Lefkowitz R.J. , Shenoy S.K. Transduction of receptor signals by beta-arrestins. Science. 2005; 308:512–517. 15845844 10.1126/science.1109237

[15] Shukla A.K. , Westfield G.H., Xiao K., Reis R.I., Huang L.Y., Tripathi-Shukla P., Qian J., Li S., Blanc A., Oleskie A.N. et al. Visualization of arrestin recruitment by a G-protein-coupled receptor. Nature. 2014; 512:218–222. 25043026 10.1038/nature13430 PMC4134437

[16] Latorraca N.R. , Wang J.K., Bauer B., Townshend R.J.L., Hollingsworth S.A., Olivieri J.E., Xu H.E., Sommer M.E., Dror R.O Molecular mechanism of GPCR-mediated arrestin activation. Nature. 2018; 557:452–456. 29720655 10.1038/s41586-018-0077-3 PMC6294333

[17] Cahill T.J. 3rd , Thomsen A.R., Tarrasch J.T., Plouffe B., Nguyen A.H., Yang F., Huang L.Y., Kahsai A.W., Bassoni D.L., Gavino B.J. et al. Distinct conformations of GPCR-beta-arrestin complexes mediate desensitization, signaling, and endocytosis. Proc. Natl. Acad. Sci. U.S.A. 2017; 114:2562–2567. 28223524 10.1073/pnas.1701529114 PMC5347553

[18] Latorraca N.R. , Masureel M., Hollingsworth S.A., Heydenreich F.M., Suomivuori C.M., Brinton C., Townshend R.J.L., Bouvier M., Kobilka B.K., Dror R.O How GPCR phosphorylation patterns orchestrate arrestin-mediated signaling. Cell. 2020; 183:1813–1825. 33296703 10.1016/j.cell.2020.11.014 PMC7901245

[19] Puca L. , Brou C. Alpha-arrestins - new players in Notch and GPCR signaling pathways in mammals. J. Cell Sci. 2014; 127:1359–1367. 24687185 10.1242/jcs.142539

[20] Cherezov V. , Rosenbaum D.M., Hanson M.A., Rasmussen S.G., Thian F.S., Kobilka T.S., Choi H.J., Kuhn P., Weis W.I., Kobilka B.K. et al. High-resolution crystal structure of an engineered human beta2-adrenergic G protein-coupled receptor. Science. 2007; 318:1258–1265. 17962520 10.1126/science.1150577 PMC2583103

[21] Velazhahan V. , Ma N., Pandy-Szekeres G., Kooistra A.J., Lee Y., Gloriam D.E., Vaidehi N., Tate C.G. Structure of the class D GPCR Ste2 dimer coupled to two G proteins. Nature. 2021; 589:148–153. 33268889 10.1038/s41586-020-2994-1 PMC7116888

[22] Liang Y.L. , Belousoff M.J., Fletcher M.M., Zhang X., Khoshouei M., Deganutti G., Koole C., Furness S.G.B., Miller L.J., Hay D.L. et al. Structure and dynamics of adrenomedullin receptors AM1 and AM2 reveal key mechanisms in the control of receptor phenotype by receptor activity-modifying proteins. ACS Pharmacol. Transl. Sci. 2020; 3:263–284. 32296767 10.1021/acsptsci.9b00080 PMC7155201

[23] Esguerra M. , Siretskiy A., Bello X., Sallander J., Gutierrez-de-Teran H. GPCR-ModSim: a comprehensive web based solution for modeling G-protein coupled receptors. Nucleic Acids Res. 2016; 44:W455–W462. 27166369 10.1093/nar/gkw403 PMC4987938

[24] Schneider J. , Ribeiro R., Alfonso-Prieto M., Carloni P., Giorgetti A. Hybrid MM/CG webserver: automatic set up of molecular mechanics/coarse-grained simulations for human G protein-coupled receptor/ligand complexes. Front. Mol. Biosci. 2020; 7:576689. 33102525 10.3389/fmolb.2020.576689 PMC7500467

[25] Sandal M. , Duy T.P., Cona M., Zung H., Carloni P., Musiani F., Giorgetti A. GOMoDo: a GPCRs online modeling and docking webserver. PLoS One. 2013; 8:e74092. 24058518 10.1371/journal.pone.0074092 PMC3772745

[26] Kooistra A.J. , Mordalski S., Pandy-Szekeres G., Esguerra M., Mamyrbekov A., Munk C., Keseru G.M., Gloriam D.E. GPCRdb in 2021: integrating GPCR sequence, structure and function. Nucleic Acids Res. 2021; 49:D335–D343. 33270898 10.1093/nar/gkaa1080 PMC7778909

[27] Rodriguez-Espigares I. , Torrens-Fontanals M., Tiemann J.K.S., Aranda-Garcia D., Ramirez-Anguita J.M., Stepniewski T.M., Worp N., Varela-Rial A., Morales-Pastor A., Medel-Lacruz B. et al. GPCRmd uncovers the dynamics of the 3D-GPCRome. Nat. Methods. 2020; 17:777–787. 32661425 10.1038/s41592-020-0884-y

[28] Sehnal D. , Bittrich S., Deshpande M., Svobodova R., Berka K., Bazgier V., Velankar S., Burley S.K., Koca J., Rose A.S. et al. Mol* Viewer: modern web app for 3D visualization and analysis of large biomolecular structures. Nucleic Acids Res. 2021; 10.1093/nar/gkab314. PMC8262734 33956157

[29] Hoksza D. , Gawron P., Ostaszewski M., Schneider R. MolArt: a molecular structure annotation and visualization tool. Bioinformatics. 2018; 34:4127–4128. 29931246 10.1093/bioinformatics/bty489 PMC6247942

[30] Rose A.S. , Hildebrand P.W. NGL Viewer: a web application for molecular visualization. Nucleic Acids Res. 2015; 43:W576–W579. 25925569 10.1093/nar/gkv402 PMC4489237

[31] Rose A.S. , Bradley A.R., Valasatava Y., Duarte J.M., Prlic A., Rose P.W. NGL viewer: web-based molecular graphics for large complexes. Bioinformatics. 2018; 34:3755–3758. 29850778 10.1093/bioinformatics/bty419 PMC6198858

[32] Lazaridis T. Effective energy function for proteins in lipid membranes. Proteins. 2003; 52:176–192. 12833542 10.1002/prot.10410

[33] Phillips J.C. , Hardy D.J., Maia J.D.C., Stone J.E., Ribeiro J.V., Bernardi R.C., Buch R., Fiorin G., Henin J., Jiang W. et al. Scalable molecular dynamics on CPU and GPU architectures with NAMD. J. Chem. Phys. 2020; 153:044130. 32752662 10.1063/5.0014475 PMC7395834

[34] Lazaridis T. , Karplus M. Effective energy function for proteins in solution. Proteins. 1999; 35:133–152. 10223287 10.1002/(sici)1097-0134(19990501)35:2<133::aid-prot1>3.0.co;2-n

[35] Ryckaert J.-P.C. , Berendsen H.J.C Numerical integration of the cartesian equations of motion of a system with constraints: Molecular dynamics of n-alkanes. J. Comput. Phys. 1977; 23:327–341.

[36] Brooks B.R. , Brooks C.L. 3rd, Mackerell A.D. Jr, Nilsson L., Petrella R.J., Roux B., Won Y., Archontis G., Bartels C., Boresch S. et al. CHARMM: the biomolecular simulation program. J. Comput. Chem. 2009; 30:1545–1614. 19444816 10.1002/jcc.21287 PMC2810661

[37] Lomize M.A. , Pogozheva I.D., Joo H., Mosberg H.I., Lomize A.L. OPM database and PPM web server: resources for positioning of proteins in membranes. Nucleic Acids Res. 2012; 40:D370–D376. 21890895 10.1093/nar/gkr703 PMC3245162

